# Optimisation of ketamine‐xylazine anaesthetic dose and its association with changes in the dendritic spine of CA1 hippocampus in the young and old male and female Wistar rats

**DOI:** 10.1002/vms3.936

**Published:** 2022-09-12

**Authors:** Narges Sotoudeh, Mohammad Reza Namavar

**Affiliations:** ^1^ Department of Anatomical Sciences School of Medicine, Shiraz University of Medical Sciences Shiraz Iran; ^2^ Histomorphometry and Stereology Research Center Shiraz University of Medical Sciences Shiraz Iran; ^3^ Clinical Neurology Research Center Shiraz University of Medical Sciences Shiraz Iran

**Keywords:** aging, anaesthesia, CA1 region, dendritic spine, ketamine, xylazine

## Abstract

**Background:**

A combination of ketamine‐xylazine (K‐X) is frequently used for anaesthesia in rats. Sex and age affect this cocktail dosage. Ketamine causes a hypnotic effect by blocking NMDA receptors located on the dendritic spine of the CA1 region.

**Objectives:**

The present study aimed to find the optimal dosage of K‐X and its association with the changes in dendritic spine number of the CA1 region for aged and young rats of both sexes.

**Methods:**

We injected 150–4 mg/kg of K‐X in young and 100–2 mg/kg in aged Wistar rats intraperitoneally and recorded the onset time and duration of anaesthesia and death percentage. Then, animals were sacrificed, brains removed, cut and after Golgi‐Cox staining, the total number of dendritic spines on CA1 was estimated.

**Results:**

The findings showed that the onset time of anaesthesia lasted longer and its duration lasted shorter, and the number of mature spines decreased with aging, but sex caused no significant effect. The death percentages in young groups comprise 20% and in the aged groups were lower: 5% in males and 0.0% in females.

**Conclusions:**

It seems 100–2 mg/kg of K‐X is an optimal dose in aged rats and retains an association with reduction of the mature dendritic spine of CA1.

## Graphical abstract

- The K‐X dosage of 100‐2 mg/kg may be an optimal anesthesia dose in old Wistar rats.
- The K‐X dosage of 150‐4 mg/kg shouldn't be recommended for young Wistar rats.
- The number of mature dendritic spine of the CA1 decreased with aging in rats.
- Mature spine density in the CA1 has correlation with the anesthesia time in old rats.

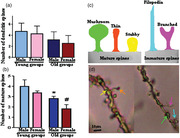

## 1 INTRODUCTION

The combination of ketamine and xylazine is widely used in anaesthesia for laboratory animals (Richardson & Flecknell, 2005). Ketamine is a glutamate n‐Methyl‐D‐aspartate antagonist that causes proper anaesthesia and analgesia (Whipe et al., 1982). There are some advantages, such as different routes of administration, high safety and the possibility of being combined with other drugs such as xylazine (Gaertner, 2008). Xylazine is an α2‐adrenergic agonist with analgesic, sedative and muscle‐relaxant properties (Giroux et al., 2015). Ketamine‐xylazine (K‐X) combination is given collectively by numerous ways of administration, such as intraperitoneal and intramuscular, providing enough surgical anaesthesia time and also it is good pain relief in rats (Buitrago et al., 2008). Administered anaesthetic doses are very different in the literature, with 40–260 mg/kg for ketamine and 5–39 mg/kg for xylazine in intramuscular injection and 75–100 mg/kg for ketamine and 10 mg/kg for xylazine in intraperitoneal injection (Dittmar et al., 2004).

Furthermore, ketamine recently has been used as a new treatment for mental disorders including depression and post‐traumatic stress disorder (Nicol & Morton, 2020). Nevertheless, some negative side effects of K‐X such as cardiac depression, bradycardia, hypotension (Buitrago et al., 2008), and respiratory depression have been reported (Schwenke & Cragg, 2004). High doses of xylazine have been shown to likely cause pulmonary oedema and death in Sprague‐Dawley rats (Amouzadeh et al., 1993; Amouzadeh et al., 1991; Kanniappan & Ramaswamy, 1979). Some factors like age and sex can affect drug distribution and pharmacokinetics (Song et al., 2012; Veilleux‐Lemieux et al., 2012). Aging because of subclinical inflammation, obesity and less activity could affect drug metabolism (Mézière et al., 2013). Also, it seems that the pharmacokinetics of ketamine and xylazine (lipophilic drugs) changes with aging because of the increase in fat deposits. Further, it has reported the high mortality rate of old mice after injection of a standard dose of K‐X in laboratory veterinary medicine (Giroux et al., 2015). For example, Schuetze et al. (2019) anaesthetised young C57BL/6‐N mice (2 months) and aged mice (26 months) by injection of 2 mg ketamine and 0.2 mg xylazine, and they reported a dead percentage of 15.4 and 0.0 in aged and young mice, respectively. A recent study reported that a high dose of ketamine (125 mg/kg) and xylazine (10 mg/kg) caused cardiac depression and pulmonary oedema in the 6‐ and 12‐month‐old Sprague Dawley rats, too (Giroux et al., 2015). Thereby, the aged individuals react differently to anaesthetics due to the age‐related alternation of pharmacodynamics features and decline in the organ function (Buitrago et al., 2008; Giroux et al., 2015).

In terms of sex differences, Schuetze et al. (2019) reported no differences in death rate and living in aged mice between both sexes. Meanwhile, Hohlbaum et al. (2018) reported a shorter anaesthesia duration in the female C57BL/6JRj mice after injection of 80 mg/kg ketamine and 16 mg/kg xylazine.

The exact mechanisms of action of ketamine in anaesthesia are not clear yet, while it is reported that, the anaesthetic drugs such as ketamine induce general anaesthesia by blockage of excitatory glutamatergic neurotransmission in the CNS by the development of analgesia and unconsciousness. Enhancement of GABAergic neurotransmission induces unconsciousness; this confirms that NMDA receptors that are a major component of glutamatergic neurotransmission can play an important role in the mechanisms of anaesthesia (Petrenko et al., 2014). For instance, it is reported that NMDA knockout animals were completely resistant to ketamine (Schuetze et al., 2019). NMDA receptors are a subtype of ionotropic glutamate receptors that express in somata and dendritic spines of pyramidal neurons and interneurons (Markham et al., 2005; Roberts et al., 2010). Studies also suggest that ketamine may trigger signaling events via inhibition of NMDA receptors in the hippocampus by synaptic transmission (Zorumski et al., 2016). Also, NMDA receptors are mostly expressed in the hippocampal regions, especially the CA1 region (Strange et al., 2014). Besides, it is reported that ketamine suppresses the connection from the medial prefrontal cortex to CA1 through NMDA receptors in rats (Moran et al., 2015).

So, any changes in spine density and morphology in neurons of the CA1 region cause changes in the NDMA receptor and consequently the hypnotic effect of ketamine and anaesthesia. This study aimed to optimise the appropriate dosage of K‐X in old and young Wistar rats of both sexes and also investigate the association of these dose differences and possible changes in spine density and maturity of the CA1 region of the hippocampus in rats. Therefore, we aimed to assess the percentage of death, start time of anaesthesia, and its duration after injection of K‐X dosage based on a pilot study, as well as the changes in spine density and maturity of pyramidal neurons of CA1 area by stereological methods.

## 2 MATERIALS AND METHODS

### 2.1 Animals and group design

Thirty‐two male and 24 female Wistar rats were purchased from the Institute of Animal Science Laboratory of Tehran (Medzist, Tehran, Iran). The animals were assigned to the young (2‐ to 3‐month‐old) male (*n* = 16) and female (*n* = 12), and old (18‐ to 20‐month‐old) male (*n* = 16) and female (*n* = 12) groups. The classification of animals into young and old rats was based on previous studies (Sengupta, 2013; Yang et al., 2018). Animals were maintained under a standard 12–12 h light‐dark cycle at room temperature (25 ± 2°C) with normal humidity. Food and water were available ad libitum. All animal procedures were approved by the local Ethical Committee of SUMS (SUMS, Shiraz, Iran, Ethic code: IR.SUMS.REC.1397.77) and were conducted following the National Institutes of Health's Guide for Care and Use of Laboratory Animals and the Animal Research: Reporting in Vivo Experiments (ARRIVE) Guidelines.

### 2.2 Treatment

In the pilot study, two different doses of K‐X were selected based on previous research (Buitrago et al., 2008; Dittmar et al., 2004; Giroux et al., 2015); in young animals, the K‐X dosage of 100–4 mg/kg and 150–4 mg/kg was injected intraperitoneally in 10 rats (3 males and 2 females for each dose) and old animals and the K‐X dosage of 80–2 mg/kg and 100–2 mg/kg was injected intraperitoneally in 10 rats (three males and two females for each dose). Then, ataxia time, negative withdrawal reflex time and anaesthesia duration were estimated. Based on the result of these anaesthesia parameters (Tables 1 and 2), only the K‐X dose of 150–4 mg/kg in young and 100–2 mg/kg in old rats induced an anaesthetic level without withdrawal reflex at the early time points (15 and 30 min). Therefore, we used these doses of ketamine and xylazine in the main study. In the main study, two different regimes of K‐X were considered for young and old groups. Young rats (male = 10, female = 8) received 150 mg/Kg ketamine (Medistar, Ascheberg, Germany) and 4 mg/kg xylazine (Riemser, Greifswald, Germany), while the old animals (male = 10, female = 8) received 100 mg/Kg ketamine and 2 mg/kg xylazine. The animals were manually restrained and based on their weight, a single dose of K‐X combination was intraperitoneally injected into the lower quadrant of the animal's abdomen. Finally, the mean time of the onset of anaesthesia, duration time of anaesthesia and death percentage were estimated.

**TABLE 1:** Time of ataxia, negative withdrawal reflex and anaesthesia duration (min) in young Wistar rats after injection of two different ketamine‐xylazine (K‐X) dosages of 100–4 mg/kg and 150–4 mg/kg

K‐X dosage	Animals	Ataxia time (min)	Negative withdrawal reflex (min)	Anaesthesia duration (min)
100–4 mg/kg	Rat 1 (M)	2	10	10
	Rat 2 (M)	–	–	–
	Rat 3 (M)	2	–	–
	Rat 4 (F)	4	–	–
	Rat 5 (F)	3.30	12	15
150–4 mg/kg	Rat 1 (M)	2	8	35
	Rat 2 (M)	3	9	30
	Rat 3 (M)	1.30	10	40
	Rat 4 (F)	2.20	10	30
	Rat 5 (F)	1.50	9	Death

–, ataxia or negative withdrawal reflex were not observed; F, female; M, male.

**TABLE 2:** Time of ataxia, negative withdrawal reflex and anaesthesia duration (min) in old Wistar rats after injection of two different ketamine‐xylazine (K‐X) dosages of 80–2 mg/kg and 100–2 mg/kg

K‐X dosage	Animals	Ataxia time (min)	Negative withdrawal reflex (min)	Anaesthesia duration (min)
80–2 mg/kg	Rat 1 (M)	2.20	12	–
	Rat 2 (M)	2	–	–
	Rat 3 (M)	3	15	10
	Rat 4 (F)	3.30	–	–
	Rat 5 (F)	3	–	–
100–2 mg/kg	Rat 1 (M)	1.30	7	20
	Rat 2 (M)	1.50	8	15
	Rat 3 (M)	2.10	9	20
	Rat 4 (F)	2.20	8	25
	Rat 5 (F)	2.30	10	20

–, ataxia or negative withdrawal reflex were not observed; F, female; M, male.

### 2.3 Evaluation of anaesthesia depth

The time after K‐X injection till incoordination and ataxia were recorded as ataxia time (Molina et al., 2015). The mean time to the onset of anaesthesia was assessed as the time after ketamine–xylazine injection until loss of the paw withdrawal reflex; it was evaluated by pressing the rat interdigital hind paw with a haemostatic forceps. The anaesthesia duration was evaluated as the period of start time to recovery of the paw withdrawal reflex (Giroux et al., 2015). To calculate the mortality, the number of dead animals in each group is divided by the total number in that group and then multiplied by 100.

### 2.4 Tissue preparation and Golgi–Cox staining procedure

On the 10th day of the experiment, three rats of each group were anaesthetised with the K‐X dosage of 100–4 mg/kg (in old rats) and 150–4 mg/kg (in young rats) and then were transcardially perfused by phosphate‐buffered saline (PBS) followed by 200–300 ml of 4% paraformaldehyde in PBS (1×, pH 7.4). The brains were removed, post‐fixed in the same fixative overnight, put in Golgi stain solution for 10 days and then transferred to a 30% sucrose solution in PBS, 4°C. After sinking, they were frozen and cut (100 μm) at –17°C to –19°C using a cryostat (Leica, CM1860, Germany). Based on previous research, the brain sections were rinsed in distilled water (5 min) and placed in the 20% Ammonia solution (10 min) and after rinsing in distilled water (5 min), dehydrated in graded ethanol and cleared by xylene (10 min). Finally, the sections were transferred to the gelatine‐coated slide and left at room temperature until dried completely (Smitha & Roopa, 2012; Sotoudeh et al., 2020).

### 2.5 Estimation of the dendritic spine density and morphology

For quantification of the total number of dendritic spine and mature spine, the microscopic images of the Gogi‐Cox stained sections were captured using a 100× oil‐immersion objective lens of the CA1 region of the hippocampus and sampled 20 pyramidal neurons which were unobscured by neighboring neurons and glia cells per group. Dendritic spines have been classified based on their morphology into mature (thin, stubby and mushroom) and immature (branched and filopodium) (Smitha & Roopa, 2012). For each selected neuron, the total number of spine and number of the mature spine was estimated at 10 μm length (which was estimated by stereological tools) located on the distal end of the 2nd‐ or 3rd‐order branch of the one dendrite with high resolution (Gillani et al., 2010; Shih et al., 2013; Sotoudeh et al., 2020).

### 2.6 Statistical analysis

The result of The Kolmogorov‐Smirnov test (alpha = 0.05) showed that bodyweight of all groups, onset time of anaesthesia, and duration of anaesthesia in young male and old female groups had normal distribution, while both onset time of anaesthesia (*p* < 0.0001) and duration of anaesthesia (*p* < 0.001) in old male and young female groups did not pass normality test. GraphPad Prism 6.0 software (www.graphpad.com; GraphPad Software, San Diego, California, USA) (Zorumski et al., 2016) (20) was carried out to perform statistical analysis and graphical presentations. The Kolmogorov‐Smirnov test was used to determine the normality of data; the one‐way ANOVA test with Tukey's post hoc test was performed for the parametric comparison (bodyweight of animals) and Kruskal‐Wallis with Dunn post hoc test for non‐parametric one (onset time of anaesthesia, duration of anaesthesia and the total number of dendrite spine). *p* Values < 0.05 were considered statistically significant.

## 3 RESULTS

### 3.1 Pilot study

#### 3.1.1 Young animals

As results have been shown in Table 1, in the dosage of 100–4 mg/kg K‐X, only two of five animals had successful anaesthesia without enough duration, while in the dose of 150–4 mg/kg K‐X, four rats showed appropriate anaesthesia duration, ataxia time and negative withdrawal reflex time. Therefore, we preferred the dosage of 150–4 mg/kg for our main study in young animals.

#### 3.1.2 Old animals

Based on the results in Table 2, in the dosage of 80–2 mg/kg, the loss of withdrawal reflex was not observed in three of five animals, and four of them were not anaesthetised with this dose of K‐X. In dosage of 100–2 mg/kg, all five animals showed ataxia and negative withdrawal reflex in acceptable time and they were anaesthetised for 15–25 min with no death. Therefore, we preferred the dose of 100–2 mg/kg for old animals in the main study.

### 3.2 Main study

#### 3.2.1 Sex differences

##### Young groups

**FIGURE 1:**
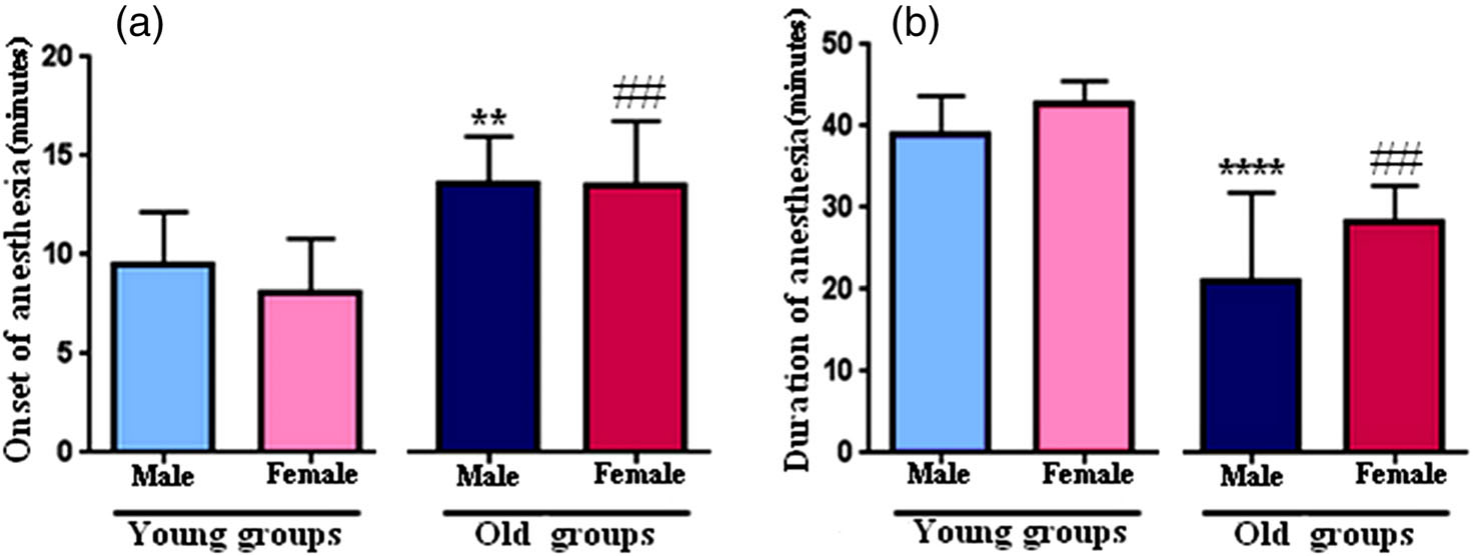
Mean ± standard deviation of the onset time of anaesthesia (a), and anaesthesia duration (b) in the young (male, female) and old (male, female) groups. **p < 0.01 and ****p < 0.0001: old male vs. young male, ^##^ *p* < 0.01: old female vs. young female.

**FIGURE 2:**
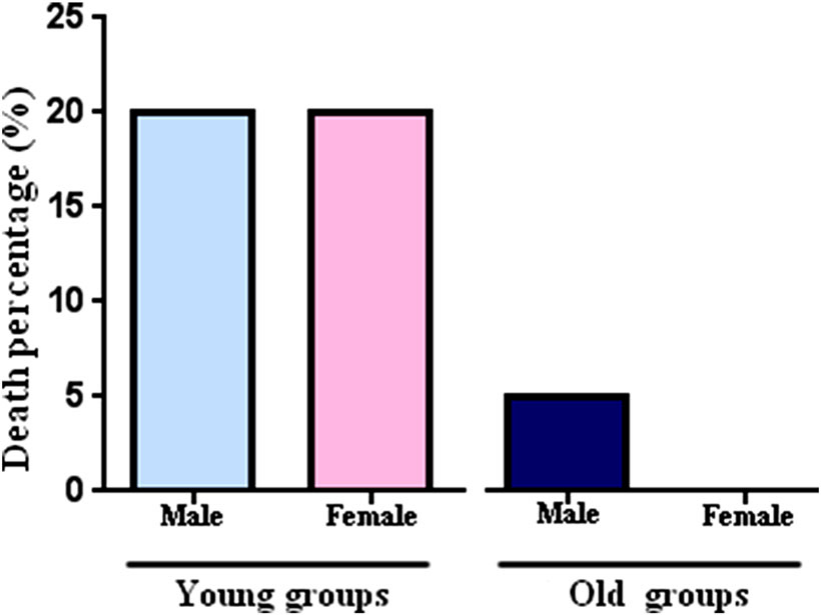
Death percentage of young (male, female) and old (male, female) animals; the young rats (male and female) had the highest percentage of death (20%) and old females the least (0.0) (*n* = 10 males, 8 females/group).

There were no significant differences in body weight between male and female rats (Table 3). The mean time to onset of anaesthesia did not show a significant difference between the groups (male: 9.4 ± 2.69, female: 8.1 ± 2.67 min, Figure 1a). Also, anaesthesia duration did not show a significant difference although it was longer in the young female than male rats (male: 38.89 ± 1.62, female: 42.86 ± 1.00 min, Figure 1b). In both groups, the death percentage was completely identical (20%, Figure 2).

**TABLE 3:** Mean ± standard deviation of body weight, ketamine (mg) and xylazine (mg) dosage per animal in the experimental groups of the main study

Group	Weight	Ketamine (mg)	Xylazine (mg)
Young male	201 ± 4.28	30.16 ± 0.64	0.76 ± 0.01
Young female	215.1 ± 13.49	32.27± 1.25	0.85 ± 0.02
Old male	476.9 ± 8.35^***,££^	47.69 ± 1.34	0.95 ± 0.02
Old female	334.6 ± 10.1^##^	33.44 ± 1.00	0.66 ± 0.02

***p < 0.001 old male vs. young male.

^##^ *p* < 0.01 old female vs. young female.

^££^ *p* < 0.01 old male vs. old female.

##### Old groups

The bodyweight of the old male animals was significantly more than the old female rats (*p* < 0.01, Table 3). There was no difference in the meantime to the onset of anaesthesia (Figure 1a) and anaesthesia duration (Figure 1b) in both sexes. However, the death percentage in males and females was different (male: 5%, female: 0.0, Figure 2).

#### 3.2.2 Age differences

Old animals had more weight than the young ones (male: *p <* 0.001, female: *p* < 0.01, Table 3). Old groups showed a longer time for the onset of anaesthesia (male: *p* < 0.01, female: *p* < 0.01, Figure 1a) and lower anaesthesia duration (male: *p* < 0.0001, female: *p* < 0.01, Figure 1b) in comparison with young animals. The death percentage in the old groups (male: 5%, female: 0.0%, Figure 2) was less than the young animals (male and female: 20%, Figure 2).

#### 3.2.3 Total number of dendritic spines

**FIGURE 3:**
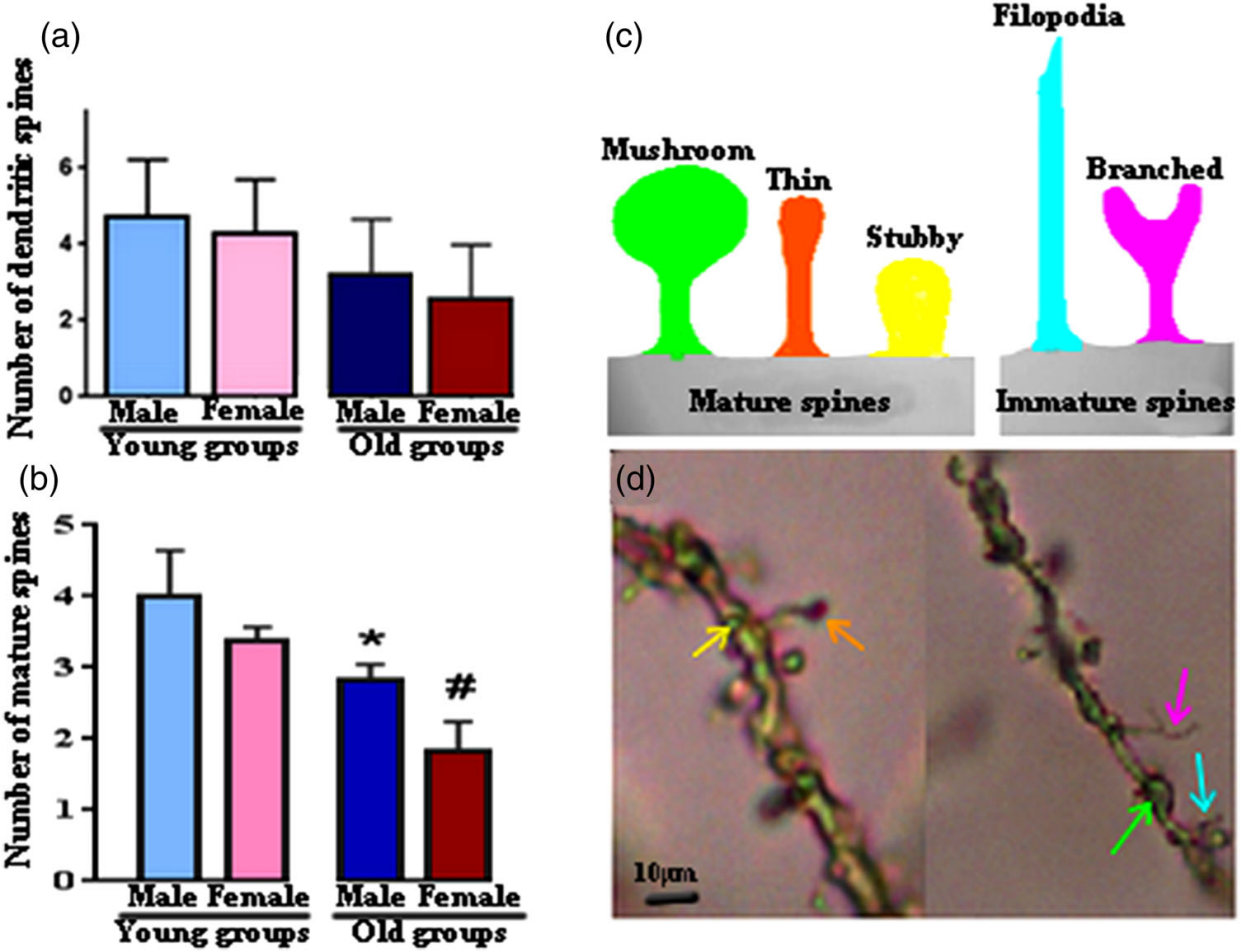
Mean ± standard deviation of the total number of the spine (a) and the number of the mature spine (b) in the CA1 region in all experimental groups. (c) Schematic of spine classification based on spine morphology; mature spine (mushroom, thin and stubby) and immature (filopodia and branched). (d) Representative microphotograph of dendritic spines on the distal end of 2nd‐ or 3rd‐order branch of dendrite on the CA1 region, the colour of the arrow is set based on c and shows the type of spine; mushroom = light green, thin = orange, stubby = yellow, filopodia = light blue, and branched = purple. *p < 0.05: old male vs. young male, #*p* < 0.05: old female vs. young female.

There was no difference in the total number of spine/10 μm on the distal end of 2nd‐ or 3rd‐order branch of the one dendrite between groups (Figure 3a), while the number of the mature spine in old groups significantly decreased in comparison with young groups (*p* < 0.05, Figure 3b).

#### 3.2.4 Correlation between the mature spine number and anaesthesia parameters

There was no correlation between the mature spine number, start time and duration time of anaesthesia in both young groups and the old male group, whereas the number of the mature spine in the old female group had a positive correlation with the duration time of anaesthesia (*r* = 0.7, *p* = 0.01).

## 4 DISCUSSION

Rats are commonly anaesthetised using a ketamine–xylazine (K‐X) cocktail (Spikes et al., 1996). This combination is considered relatively safe in rats and achieves effective analgesia, muscle relaxation and sedation (Wyatt et al., 1989). Ketamine has a hypnotic effect by blocking NMDA receptors of the CA1 region. So, in this study, we have focused on the most appropriate dose of K‐X and its association with changes in the dendritic spine of the CA1 region in the young (2–3 months) and old (18–20 months) Wistar rats in both sexes. In this manner, we assessed the mean time to onset of anaesthesia, anaesthesia duration and death percentage between these groups after injection of K‐X dosage of 150–4 mg/kg in young and 100–2 mg/kg in old rats. Also, we estimated the total number of spines and mature spines in the dendrites of the CA1 pyramidal neurons.

Our result indicated no difference in the meantime to the onset of anaesthesia in young groups between male and female rats (Figure 1a). This finding is in agreement with a previous study that injected a K‐X dosage of 191.25–4.25 mg/kg in the male and female mice (12 weeks) intraperitoneally and found the onset time of surgical anaesthesia in the female mice was not different from the male ones (Levin‐Arama et al., 2016). Furthermore, Hohlbaum et al. (2018) injected 80 mg/kg ketamine and 16 mg/kg xylazine in 33 adult female and 31 adult male C57BL/6JRj mice (10–13 weeks) and found that the time of anaesthesia induction in males was not significantly different from the females. It seems that high doses of K‐X cause fast anaesthesia with no gender difference. Furthermore, to the best of our knowledge, there is no information regarding the comparison of the mean time to onset of anaesthesia in aged and young animals in recent research.

In the current study, the duration of anaesthesia was significantly higher in young animals than in old ones. At both ages, the female rats experienced longer anaesthesia duration than males although this difference was not statistically significant (Figure 1b). Giroux et al. have recently used rats at different ages of 3, 6 and 12 months, which received 125 mg/kg of ketamine and 10 mg/kg xylazine intraperitoneally and were followed at different times of 5, 15, 30 and 45 min after injection. Their results showed that higher doses of K‐X increased the duration of anaesthesia. However, cardiac depression and pulmonary oedema were observed in the 6‐ and 12‐month‐old rats (Giroux et al., 2015). These findings, like our results, suggest that a higher dose of K‐X is associated with prolonging the duration of anaesthesia and possibly increasing mortality in young rats. Also, it indicates that the optimal dose of K‐X in aged animals must be lower than in young ones.

In another study, 125 mg/kg ketamine and 10 mg/kg xylazine were injected in the young (2–3 months) and old Sprague‐Dawley rats (24 months). Its results significantly showed longer anaesthesia duration in the old rats than in young ones (Veilleux‐Lemieux et al., 2013). Thus, it seems that regardless of the mortality rate and toxicity side effects, a high dosage of ketamine and xylazine is accompanied by an increase in the duration of anaesthesia, and both ketamine and xylazine have dose‐dependent side effects. Also, it is reported that a dosage of 80 mg/kg ketamine and 16 mg/kg xylazine in the adult female and male C57BL/6JRj mice (10–13 weeks) resulted in non‐significant longer anaesthesia duration in the female than males (Hohlbaum et al., 2018). This report is in line with our finding that there is no significant difference in anaesthesia duration between males and females of the same age.

In our study, both young groups (male and female) had the same death percentage (20%), while the old animals had lower mortality. Surprisingly, old females had the lowest mortality rate, as shown in Figure 2. Furthermore, we could not determine the exact cause of mortality. However, hypothermia or drug hypersensitivity, respiratory obstruction due to an unfavourable positioning, and route injection of medications may be involved.

There are some controversial data concerning the mortality and anaesthesia parameters related to K‐X used in recent research. Regarding the young animal mortality, Levin‐Arama et al. (2016) reported that injection of 191.25–4.25 mg/kg of K‐X in mice (3 months) caused 30% mortality in females and 3% in males, while in our study, there was no difference in the mortality rate between young male and female rats. These different results could be related to the difference in animal strain (Wistar rat in our study and mice in Levin‐Arama study) and the difference in the dosage of K‐X (150–4 mg/kg in our study and 191.25–4.25 mg/kg in Levin‐Arama study). Also, this difference could be associated with other reasons like body weight, sex hormones, plasma corticosteroids and hepatic enzymes (Ishizaka et al., 2004).

Regarding the mortality rate and age differences, Schuetze et al. anaesthetised C57BL/6‐N mice at the age of two months (young mice) and 26 months (aged mice) by intraperitoneal injection of 2 mg ketamine and 0.2 mg xylazine. They reported a mortality rate of 15.4% in the old mice and 0.0% in young ones, and no differences between the deceased and surviving aged mice concerning their sex (Schuetze et al., 2019). This is in line with our results regarding no differences between the sex of animals in the mortality rate in young animals and a low difference in aged animals. However, it is in contrast with the death percentage in the aged rats in our study. There is a possible explanation for the high mortality rate in the current study; high mortality in young animals could be attributed to the low body mass and route of administration. It seems that in intraperitoneal administration, K‐X distributes fast and causes cardiac‐pulmonary arrest. Additional studies are required to understand the relationship between anaesthetic depth and the concentrations of drugs in the nervous tissue and plasma. It seems that intramuscular or subcutaneous injection of K‐X for young rats is probably more efficient. Besides, this ratio of two drugs may not be appropriate, and probably xylazine does not have an adequate sedative in combination with ketamine for young Wistar rats (Molina et al., 2015).

Regarding the high mortality with a high dosage of K‐X, a previous study reported that xylazine causes hypoxia and acidosis because of airway constriction (Wixson et al., 1987). It has been also reported that ketamine–xylazine ventilation in rats decreased PO_2_ and O_2_ saturation and arterial oxygen content, and it reduced brain oxygenation in rats (Lei et al., 2001).

It is clear that aging causes physiological and morphological changes in the body organs such as the brain. Therefore, we assessed the changes in the dendritic spine in the CA1 region, where the NMDA receptor is located. In our study, the total number of spines between the young and old groups is stable, while the number of mature spines in the CA1 region decreased significantly in the old groups. In this term, Markham et al. (2005) reported no change in the spine density of the CA1 region, in either sex in young (3–5 months) and old (19–22 months) rats, but they observed a 25% decrease in the CA3 region. In addition, Wasowicz et al. (1996) evaluated the spine density of apical dendritic of CA1 in 3‐, 5‐ and 7‐ month‐old mice and reported that 7‐month‐old mice had a lower number of spine than 5‐month‐old. It seems that different brain areas exhibit differing levels of synaptic turnover during development (Roberts et al., 2010).

In addition, spines are substrates for synaptic transmission and they can affect the possible contacts between neurons (Tackenberg et al., 2009). It is reported that changes in the shape and size of dendritic spines are correlated with the strength of excitatory synaptic (Hotulainen & Hoogenraad, 2010). So, it seems that in old rats because of the lower mature spine and might less NMDA receptors, the dosage of K‐X for induction of anaesthesia is lower than for young ones. Certainly, more studies on molecular mechanisms are needed.

## 5 CONCLUSION

In conclusion, this study shows that the high intraperitoneal dosage of K‐X (150–4 mg/kg) should not be recommended for anaesthesia in young (2–3 months) Wistar rats, and the intraperitoneal injection of 100–2 mg/kg of K‐X dose could be the optimal dose and route in the male and female aged (18–20 months) Wistar rats. Also, it seems that the reduction of the mature spines of CA1 in old rats has associations with a lower dose of K‐X dosage and less duration anaesthesia time.

## AUTHOR CONTRIBUTIONS

Narges Sotoudeh: Investigation; methodology; writing‐original draft. Mohammad Reza Namavar: Funding acquisition; project conception, design, and supervision; data analysis; writing‐review & editing. Both authors read and approved the final manuscript.

## ETHICAL STATEMENT

It is emphasised that this article has not been submitted to any other journals for publication. It is acknowledged that both authors have contributed significantly and agree with the contents of the manuscript. The authors are affiliated with Shiraz University of Medical Sciences is an academic place of education and research. Animal procedures were conducted under the National Institutes of Health's Guide for Care and Use of Laboratory Animals and the Animal Research: Reporting in Vivo Experiments (ARRIVE) guiding principle and approved by the Ethics Committee of Shiraz University of Medical Sciences (SUMS, Ethical code: IR.SUMS.REC.1397.77).

## CONFLICT OF INTEREST

The authors declare they do not have any known competing financial interests or personal relationships that could have appeared to influence the work reported in this paper.

6

### PEER REVIEW

The peer review history for this article is available at https://publons.com/publon/10.1002/vms3.936.

## Data Availability

No.
